# Does Previous Hip Surgery Effect the Outcome of Tönnis Triple Periacetabular Osteotomy? Mid-Term Results

**DOI:** 10.1097/MD.0000000000003050

**Published:** 2016-03-11

**Authors:** Mehmet Nuri Konya, Bahattin Kerem Aydın, Timur Yıldırım, Hakan Sofu, Sarper Gürsu

**Affiliations:** From the Department of Orthopedics and Traumatology, Selcuk University, Konya (BKA); Baltalimanı Bone Joint Diseases Research and Education Hospital, İstanbul (TY, SG); Department of Orthopedics and Traumatology, Erzincan University, Erzincan (HS), and Department of Orthopedics and Traumatology, Kocatepe University, Afyon (MNK), Turkey.

## Abstract

Hip dysplasia (HD) is 1 of the major reasons of coxarthrosis. The goal of the treatment of HD by Tönnis triple pelvic osteotomy (TPAO) is to improve the function of hip joint while relieving pain, delaying and possibly preventing end-stage arthritis. The aim of this study is to compare the clinical and radiological results of TPAO to determine if previous surgery has a negative effect on TPAO.

Patients operated with TPAO between 2005 and 2010, included in this study. Patients divided into 2 groups: primary acetabular dysplasia (PAD) and residual acetabular dysplasia (RAD). Prepostoperatively, hip range of motion, Harris hip score (HHS), Western Ontario and McMaster Universities Osteoarthritis Index (WOMAC) hip score, visual analog scores (VAS), impingement tests, and also the presence of Trendelenburg sign (TS) were investigated for clinical evaluation. For radiological analysis pre–postoperative, anterior–posterior (AP) pelvis and faux profile radiographs were used. Acetabular index, lateral center edge (LCE) angle, and Sharp angles were measured by AP pelvis; anterior center edge (ACE) angle were measured by faux profile radiography. All the clinical and radiological data of the groups were analyzed separately for the pre–postoperative scores also the amount of improvement in all parameters were analyzed.

SPSS20 (SPSS Inc., Chicago, IL) was used for statistical analysis. Wilcoxon test, McNemar test, paired *t* tests, and Mann–Whitney *U* tests were used to compare the groups. *P* < 0.05 were defined as statistically significant.

Study included 27 patients: 17 patients were in PAD and 10 patients were in RAD. The mean follow-up period was 6.2 years (5.2–10.3 years). In all patients, the radiological and the clinical outcomes were better after TPAO except the flexion of the hip parameter. When the patient groups were evaluated as pre–postoperatively, more statistically significant parameters were found in the PAD group when compared with RAD group. Extension, impingement, TS, VAS, HHS, WOMAC score parameters in clinical outcome and LCE, ACE, Sharp angle, coverage ratio in radiological results were significantly better in PAD group postoperatively but in RAD group; only extension, VAS, HHS, and WOMAC parameters were clinically and LCE and Coverage ratio were significantly different compared with the preoperative measurements. The change of the parameters that used for the evaluation of clinical and radiological results did not show a significant difference between groups.

Our data suggest that TPAO can be performed on patients with HD for both groups. Although there were fewer parameters which changed significantly after TPAO in RAD patients; the improvement of radiological and clinical results was similar for groups. Further long-term follow-up studies with large number of patients are needed to determine the proper results of TPAO.

## INTRODUCTION

Hip dysplasia (HD) is still 1 of the major reasons of secondary osteoarthritis of the hip.^1,2^ Patients are frequently presented in adolescence or young adulthood with prearthritic hip symptoms related to acetabular dysplasia.^3^ When left untreated, this structural abnormality can cause hip degeneration and eventual end-stage disease.^4^ The goal of corrective surgery in the treatment of HD is to improve the function while relieving pain, delaying and possibly preventing the end-stage arthritis.^5,6^ Different kinds of periacetabular osteotomies including innominate osteotomies,^7–11^ juxta-articular triple osteotomies,^12^ periacetabular osteotomies,^13^ and spherical osteotomies^14,15^ have been described to solve this problem. Tönnis triple pelvic osteotomy (TPAO) is 1 of the popular surgical osteotomies described and are still used since 1978.^12^

Tönnis triple osteotomy technique differs from other periacetabular osteotomies mainly with the additional ischial osteotomy. The osteotomies of ischium, pubis, and ilium are close enough to the hip joint to allow satisfactory rotation of the acetabulum.^16^ There are many papers about the TPAO surgery reporting improved femoral head coverage, decreased acetabular inclination, and also an improvement in hip pain and function scores.^17–19^

Tönnis periacetabular osteotomy (TPAO) can be applied to patients who have primary acetabular dysplasia (PAD) and also to the patients who had previous reconstructive hip surgeries. Hips with prior surgery procedures can be more challenging to treat regarding the scar formations, muscular imbalance, and bone healing problems secondary to the previous operations. According to our knowledge that there are plenty number of paper about comparing the results of the patients who were operated primarily and who had previous reconstructive surgery with other type of periacetabular ostetomy (PAO) called Bernese Osteotomy, but we could not find any articles about Tönnis type PAO.^9,10,20–24^

Regarding this, a study was planned to compare the results of TPAO between the primary cases and the patients who had previous reconstructive surgery for acetabular dysplasia. The aim of this study is to compare the clinical and radiological results of TPAO to determine whether if the previous surgery has a negative effect on TPAO.

## MATERIALS AND METHODS

Between 2005 and 2010, TPAO was performed for 64 patients with acetabular dysplasia in our institute. The clinical records of these patients were reviewed. The review of the data included in this retrospective study was approved by the local institutional board.

Patients who were operated for only PAD or residual acetabular dysplasia (RAD) without any other disease were included in this study. The patients were selected among being without any rheumatologic or septic arthritis, to have more than 80° of hip flexion and 45° abduction–adduction ranges, lateral center edge (LCE) angle <20°, acetabular angle more than 40°, acetabular index (AI) more than 10°, Coverage Ratio of Femoral Head (CRFH) <75%, and anterior center edge (ACE) <20° in faux profile radiographs and also osteoarthritis of hip joint lower than grade 2 which are also the indications for TPAO. Demographic data of the patients, laterality, surgery time, and the amount of blood loss during the surgery were evaluated.

Pre and postoperatively, hip range of motion (ROM), Harris hip score (HHS), Western Ontario and McMaster Universities Osteoarthritis Index (WOMAC) hip score, visual analog scores (VAS), impingement tests, and also the presence of Trendelenburg sign (TS) were investigated for clinical evaluation.

For radiological analysis pre and postoperative, anterior–posterior (AP) pelvis and faux profile radiographs used. AI, LCE angle, and Sharp angle parameters were measured by AP pelvis radiographs; ACE angle parameters were measured by faux profile radiographs.^6^

All the clinical and radiological data of the groups were analyzed separately for the pre and postoperative scores. Also the amount of improvement in all parameters was analyzed comparing the PAD and RAD groups.

### Statistical data

SPSS 20 (SPSS Inc., Chicago, IL) was used for statistical analysis. Wilcoxon test was used to compare the groups. *P* values <0.05 were defined as statistically significant. Mann–Whitney *U* test was used for the evaluation of age and time period. Chi-squared test was used for sex distribution. Levene tests and *t* tests were used to evaluate the improvement of joint motion rate. For the evaluation of categorical dependent data, McNemar test was used. Paired *t* test was used for the evaluation of quantitative dependent data when the distribution of the data was normal and Wilcoxon test was used for the evaluation of dependent quantitative dependent data when the distribution was irregular.

## RESULTS

Of the 64 patients, 21 patients were Legg Calve Perthes disease, 8 patients were operated secondary to neurologic disease (Poliomyelitis, Cerebral Palsy, and Motor Mental retardation) and 8 patients who were lost to follow-up were excluded from this study. The demographic data of the 27 patients who included in this study were presented in Table 1. The number of the patients in PAD group (PAO) was 17 (4 male and 13 female) and RAD group was 10 (3 male and 7 female), and mean age was 24.12 (11–36) in PAD group and 20.9 (9–37) in RAD group. Surgery time was 270 min in PAD group and 328 min in RAD group and the amount of bleeding were 3.24 U for PAD groups and 2.40 U for RAD groups (Table 1).

**TABLE 1:**
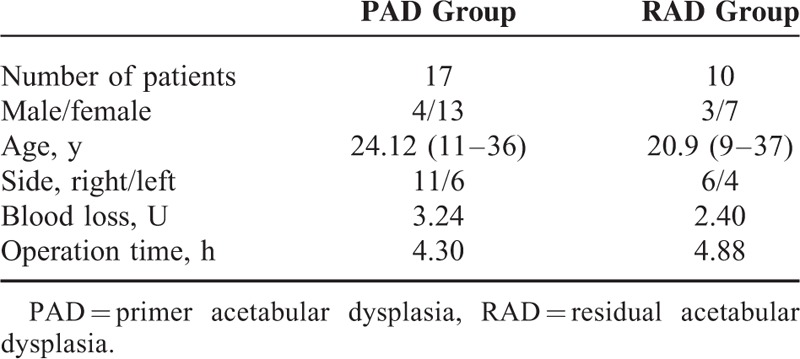
Demographic Data of the Patients

Follow-up visits were made at the 3rd, 6th, and 12th months postoperatively, and annual controls were organized.

Various surgical procedures were performed to 10 of the 27 patients prior to the triple pelvic osteotomy which included open reduction (1 patient), Salter osteotomy (8 patients), proximal femoral osteotomy (PFO) (3 patients), and combination of PFO and pelvic osteotomy (2 patients). Two patients had a history of more than 1 hip operation.

Clinical results of PAD group were shown in Table 2. According to these results in PAD group, there were only significant differences found in extension movement.

**TABLE 2:**
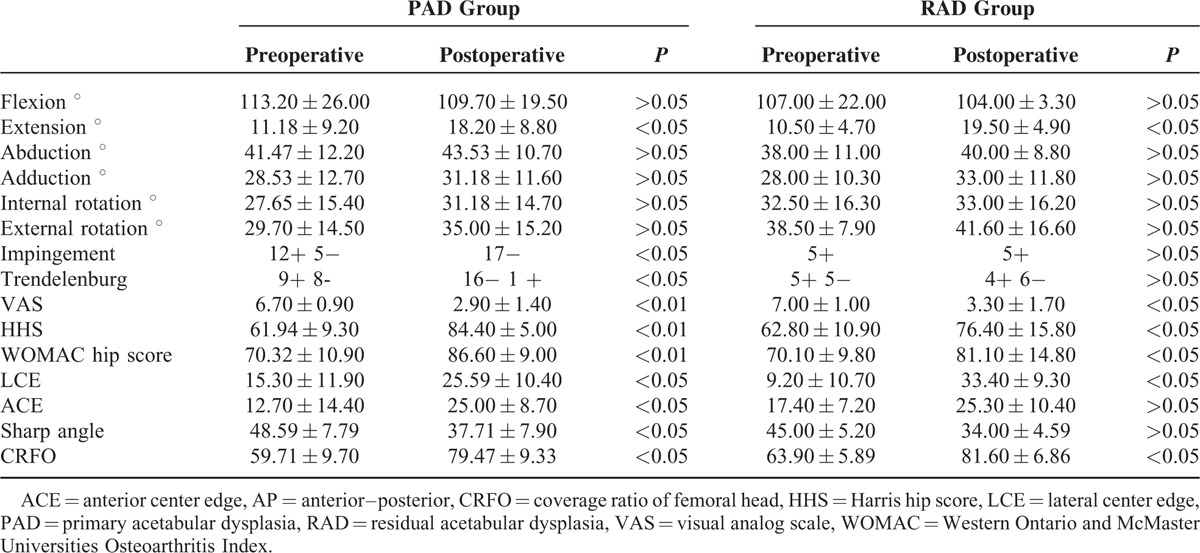
Clinical and Radiological Results of PAD and RAD Groups

LCE angle was measured preoperatively 15.3° ± 11.9° and postoperatively 25.59° ± 10.40° (*P* < 0.05). ACE angle 12.7° ± 14.4° preoperatively and 25.59° ± 10.40° postoperatively (*P* < 0.05). The mean improvement was 12.29 ± 16.99 (*P* < 0.05). Also in Sharp angle 48.59° ± 7.79° preoperatively and 37.71° ± 7.90° postoperatively (*P* < 0.05) and improvement was −10.88 ± 3.04 (*P* < 0.05). The coverage ratio was measured preoperatively 59.71 ± 9.70 and 79.47 ± 9.33 (*P* < 0.05). The improvement was 19.76 ± 7.69 (*P* < 0.05; Table 2). Trendelenburg test preoperatively were positive in 9 patients and postoperatively positive in 1 patient (*P* < 0.05). Mean HHS was changed from 61.94 ± 9.30 to 84.40 ± 5.00 after operation, the improvement was 22.52 ± 9.02 (*P* < 0.05). WOMAC scores was changed from 70.32 ± 10.90 to 86.60 ± 9.00 after operation, the improvement was 16.28 ± 10.93 (*P* < 0.05). VAS scores were used to assess patients’ pain. In PAD group, VAS scores changed from 6.7 ± 0.9 to 2.9 ± 1.4 and the improvement was 3.76 ± 1.80 (*P* < 0.01).

Of the RAD group, clinical results were presented also in Table 2 and according to these results in RAD group, there were only significant differences found in extension movement.

LCE angle was measured preoperatively 9.2° ± 10.7° and postoperatively 33.4° ± 9.3° (*P* < 0.05). ACE angle 17.4° ± 7.2° preoperatively and 25.3° ± 10.4° postoperatively (*P* < 0.05). The mean improvement was 7.9 ± 11.9 (*P* > 0.05). Sharp angle measured 45.0° ± 5.2° preoperatively and 34.00° ± 4.59° postoperatively (*P* > 0.05) and improvement was −11.00 ± 2.08 (*P* > 0.05). The coverage ratio was measured preoperatively 63.90 ± 5.89 and 81.60 ± 6.86 (*P* < 0.05). The improvement was 17.7 ± 8.2 (*P* < 0.05).

Our results showed that in PAD group all radiologic parameters, in RAD group only LCE and coverage ratio improved positively (Table 2).

Preoperatively Trendelenburg test were positive in 5 patients and postoperatively in 4 patients (*P* > 0.05). Mean HHS was changed from 62.8 ± 10.9 to 76.4 ± 15.8 after operation, the improvement was 13.50 ± 17.25 (*P* < 0.05). WOMAC scores was changed from 70.1 ± 9.8 to 81.1 ± 14.8 after operation, the improvement was 12.42 ± 9.82 (*P* < 0.05). In RAD group, VAS scores changed from 7 ± 1 to 3.3 ± 1.7 and the improvement was 3.7 ± 2.4 (*P* < 0.05).

The improvement of scores for both groups was compared in Table 3. According to improvement, there were significant differences in both groups (*P* > 0.05).

**TABLE 3:**
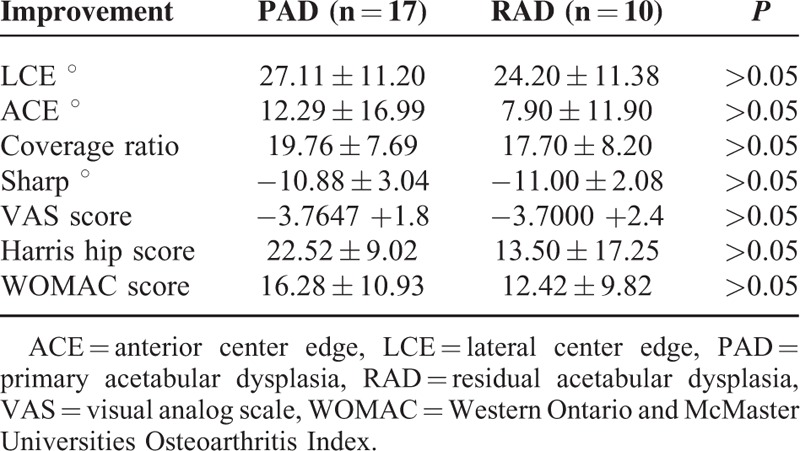
Comparison of Improvement Between PAD and RAD Groups

**FIGURE 1:**
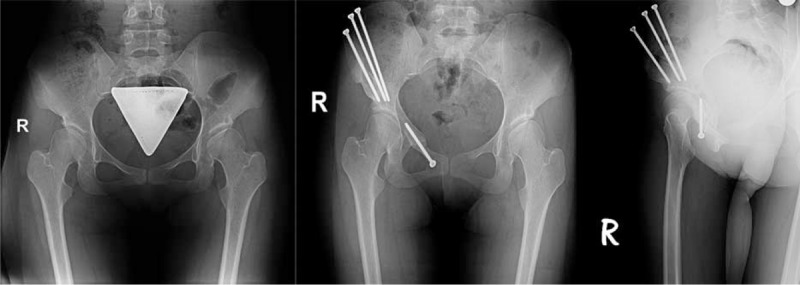
Patient 1: primary acetabular dysplasia; 21-year-old female. Preoperative and postoperative 3rd y AP and faux profile radiography.

**FIGURE 2:**
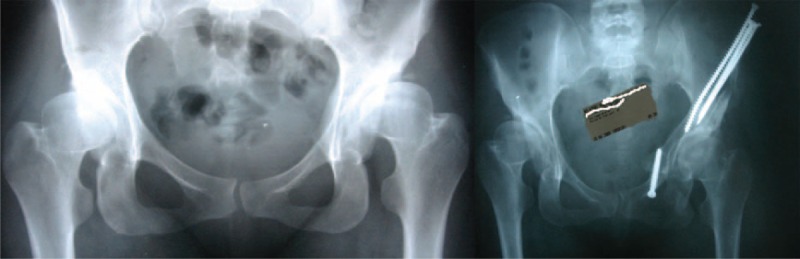
Patient 2: residual acetabular dysplasia; 30-year-old female; operated at the age of 1 for developmental dysplasia of hip; pre–postoperative radiography.

In all patients, all the radiological and the clinical outcomes were found better after TPAO except the flexion of hip parameter. When the patient groups were separately evaluated pre and postoperatively, there were more statistically significant parameters in the PAD group when compared with RAD group. Extension, impingement, Trendelenburg, VAS, HHS, and WOMAC score parameters in clinical outcome and LCE, ACE, Sharp angle, and coverage ratio in radiological results were significantly better in PAD group postoperatively (Figure 1). But in RAD group, only extension, VAS, HHS, and WOMAC parameters clinically and LCE and coverage ratio radiologically was significantly different compared with the preoperative measurements (Figure 2). The change of the parameters that used for the evaluation of clinical and radiological results did not show a significant difference between the preoperated and nonoperated groups.

The mean follow-up period was 6.2 years (5.2–10.3 years). Of the 27 patients, major complications related to the surgery were sciatic nerve neuropraxy in 1 patient who recovered 9 months later (in RAD group), wound infection in detected in the early postop period and wash out at the postoperative 13 days who healed without requiring any surgical procedure (in PAD group). None of the patients had a recurrent hip arthroplasty operation at their last follow-up visits.

## DISCUSSION

According to our results, TPAO clinical results are similar in preop and nonoperated hips. But when the number of the parameters (radiologically and clinically), which showed significant difference postoperatively, was debated there were better results in PAD group. We think that previous surgeries may affect TPAO outcome compared with the primary cases.

Pelvic osteotomies are commonly used to treat the acetabular dysplasia. Most of the patients are diagnosed in adolescence or young adulthood with prearthritic hip symptoms related to acetabular dysplasia.^3^ Some of the patients were operated during the early childhood and have some reconstructive surgeries. Periacetabular osteotomies (such as Bernese, Tönnis) are being used for surgical treatment of dysplasia in early adolescents and adulthood.^9,10,23,24^ There are limited numbers of studies evaluating the outcomes of PAO in patients who had previous reconstructive surgery and who did not. Most of the studies regard to Bernese osteotomies.^5^ Therefore, we evaluated the improvement of hip scores of the patients who were operated according to the TPAO. According to our results, TPAO surgery improves hip scores clinically and radiologically in preoperated and nonoperated patients. However, there were more clinical and radiological result parameters which significantly changed postoperatively in hips, have no previous reconstructive surgery. The clinical outcome of TPAO can be considered more successful in PAD patients.

The mean surgery time was longer, and also the amount of bleeding during the surgery was less in RAO group compared with PAO group. This was a surprising result for us as we thought that the blood loss parameter would be opposite as because of the scar tissue and adherent structures secondary to previous surgeries. But the statistical analysis did not show any significant difference may be secondary to the small number of patients in the groups.

Steel triple osteotomy reported in 1973 has probably been the most popular and has undergone substantial modifications.^25^ Tönnis invented a major modification of Steel triple osteotomies,^26^ in which the site of the ischial osteotomy, closer to the acetabulum than in Steel osteotomy, is just adjacent to the hip joint, allowing an easier rotation of the acetabulum. The contact area is superior to that of the Steel osteotomy. Bernese PAO is performed by a single incision via TPAO is performed by 3 separate incisions. This seems to be a disadvantage for TPAO, but this exposure maintains direct visualization of neurovascular structures and avoids their damage during the operation. The osteotomy used for Bernese PAO is very close to acetabulum, so this can be a disadvantage for an intraoperative intra-articular fracture. And the other disadvantage of Bernese PAO is the osteotomy passes through Y cartilage so this technique can only be performed to adult people. For these reasons, we performed TPAO to our HD patients.

All the ROM parameters except hip flexion were better postoperatively in 2 groups. The only significant difference between preoperative and postoperative ROM values in 2 groups was an extension. The amount of the change in ROM parameters did not show significant difference comparing the PAD and RAD groups (Table 3). Impingement test was negative for all the patients in PAD group postoperatively although there were 12 patients with positive impingement test preoperatively.

This parameter did not change in any of the patients in RAD group. As impingement is believed to be 1 of the reasons for hip arthrosis, this result may be a sign of high expectancy of coxarthrosis in RAD group. TS was also better postoperatively in both PAD groups. The change in RAD group was not significantly better compared with preoperative values. This result can be a result of the primary operations which can cause gluteus muscle insufficiency. We consider this is due to operation technique which requires not splitting gluteus medius muscle from the iliac bone to prevent iatrogenic gluteus medius injury. Limping is 1 of the main complaints of HD which can cause arthrosis. Having muscular imbalance and weight transfer abnormalities in lower limb can cause hip and knee problems and also lomber and sacroiliac problems. So according to our study, being in the PAD group may be an advantage for triple pelvic osteotomy. As known that, impingement and Trendelenburg signs are among the causes of coxarthrosis; this group of patients may have long survival compared with the RAD group.

The other clinical features VAS, HHS, and WOMAC scores were better postoperatively in both groups. These are the parameters commonly used for the clinical outcomes of the patients with hip problems. These parameters are the predictors of this type of surgery, are successful for HD patients. There was not any significant difference in the improvement of these parameters comparing the 2 groups.

For this study, LCE, ACE, Sharp angle, and coverage parameters were analyzed pre and postoperatively. All the radiologic parameters were changed positively in both groups. But significant changes were detected for all parameters in PAD group, only LCE and coverage ratio for RAD group. More anatomical acetabulum may be considered more normal hips. The risk for coxarthrosis may be lowered by this osteotomy. This result also can be a predictor of the success of this surgery.

The change of all the parameters was analyzed for both groups. The amount of improvement did not show any significant difference in all parameters. This result can be explained as Tönnis Triple Osteotomy has positive effects on the radiological and clinical parameters, however, having this operation.

The clinical scores in our patients improved in both preoperated and nonoperated groups. There are limited data about the PAO outcomes for the patients who had reconstructive surgery during the childhood or not.^27^ Mayo et al^28^ compared 19 hips who underwent previous surgeries with a group of patients without any previous operation. They reported the improvement of clinical scores in all patients after PAO, but they found no difference in HHS scores between the groups. Our results are similar to this study as we found improvement in all clinical scores. We also analyzed the radiological results which were also better after TPAO. Additionally we evaluated the improvement in the clinical and radiological scores in 2 groups. According to our study, the amount of improvement was similar in preoperated and nonoperated groups.

In another study, Thawrani et al^29^ studied the radiological results of PAO, who had previous surgery or not. They found no difference in radiographic deformity correction between 2 groups. Our results are also similar to Thawrani's study as we also found no difference according to the improvement of radiological outcomes in both groups. But when the groups separately evaluated in PAD group all the radiological results were significantly better in the postoperative period. The LCE and coverage ratio parameters showed significant improvement in the postoperative period. This can be secondary to the osteotomy type that used in this study which is different from Bernese Osteotomy.

There are limitations to this study. First, this is a retrospective case–control study. It is well known that prospective designs of such studies are valuable for the contribution to the literature. But in our study there is also a control group. Similar studies about this subject mainly have no control groups. Second, patients’ numbers in groups are low. Comparison of small numbered groups is difficult and also results can be less reliable to large numbered studies. Third, all operations were performed in a single center which may affect the generalization of the results. Fourth, this is a medium-term follow-up study does not give any information about long-term results. The fifth limitation of this study is the not standard surgeries of the patients who had previous hip reconstructive surgeries. The details of patients’ previous hip operations were unknown to evaluate their surgical techniques and the age of their previous operations. And the sixth limitation was age groups distributed heterogeneously but we think that the distribution of age of the patients was similar as both of the groups included younger patients, 9 and 11 years old. For the younger patient we had to perform operation due to their symptoms. Also Y cartilage was closed because of the previous operations.

Most of the previous studies have shown that improvements in radiographic parameters such as acetabular inclination and center-edge angle are achieved consistently and reliably with PAO.^5^ This is also same according to our results which show similar improvement in radiological scores.

The most commonly used scoring systems for evaluating hip function are HHS, Merle D’Aubigne score, and WOMAC. In our study, we used WOMAC and HHS for clinical outcomes. The results were similar to the previous studies showing better hip function after Tönnis type PAO. WOMAC scores improved to over 80 points substantially postoperatively and also HHSs improved to over 75 points in both PAD and RAD groups. This suggests that a major change in quality of life after PAO.

We also investigated the change in improvement among the patients who had previous hip surgeries. This point is different from the other studies in literature as we had a control group and also this is the first study about Tönnis type PAO. According to our results having previous surgery has no statistically significant effect on the improvement of radiological and clinical scores. But when the groups were analyzed separately, there were statistically significant better results achieved in PAD group.

In conclusion, our data suggest that TPAO can be performed in patients with HD both who had previous hip reconstructive surgery or not. Although, there were fewer parameters which changed significantly after TPAO in RAD patients; the improvement of radiological and clinical results were similar in RAD and PAD groups. Further long-term follow-up studies with large number of patients are needed to determine the proper results of TPAO.
